# Icterus and abdominal pain: an unexpected, rare sonographic finding in a Peruvian Emergency Department

**DOI:** 10.1186/s13089-018-0091-6

**Published:** 2018-07-12

**Authors:** Stephanie J. Doniger, Alexander Wang

**Affiliations:** 1 Department of Pediatric Emergency Medicine, St. Christopher’s Hospital for Children, Philadelphia, PA USA; 2 Department of Emergency Medicine, NYU Winthrop Hospital, Mineola, NY USA; 3 Department of Emergency Medicine, New York-Presbyterian, Brooklyn Methodist Hospital, Brooklyn, NY USA

**Keywords:** Jaundice, Point-of-care ultrasound, Choledochal cyst, Echinococcosis, Hydatid disease, Tropical medicine, Infectious disease, Right upper quadrant ultrasound, Gallbladder, Biliary tree

## Abstract

**Background:**

The use of point-of-care ultrasound (POCUS) has become increasingly important in resource-limited settings. It can rapidly diagnose both tropical infectious diseases and more common pathology at the bedside. In these practice settings, POCUS can have a significant impact on management strategies and patient care. Ultrasonography has been the gold standard for the diagnosis and staging of *Echinococcus* disease. However, even in the “classic” clinical scenario and setting, the clinician must maintain a broad differential diagnosis. Point-of-care ultrasound can be helpful in performing the rapid diagnosis and therefore direct appropriate treatment strategies based on the results.

**Case presentation:**

We present a case of a 27-year-old woman presenting to an emergency department in Peru with jaundice and abdominal pain. Initially given the region of her origin, the working diagnosis was an *Echinococcus* cyst. However, when POCUS was performed, the findings were not consistent with hydatid disease. Ultimately, surgical pathology revealed a choledochal cyst, a rare finding in adulthood.

**Conclusions:**

This case initially appears as a “classic” finding of *Echinococcus* disease. It is important for the clinician sonographer to appreciate the features consistent with *Echinococcus* cysts and distinguish from those features that are more consistent with other pathology.

**Electronic supplementary material:**

The online version of this article (10.1186/s13089-018-0091-6) contains supplementary material, which is available to authorized users.

## Background

Point-of-care ultrasound (POCUS) was first introduced in the emergency department in the 1980s. Since that time, its use has become more widespread, extending across other subspecialties and into other practice environments. Point-of-care ultrasound can be particularly useful in resource-limited practice settings, such as in the developing world. There are often significant delays and prohibitive costs associated with advanced imaging studies, such as computerized tomography and magnetic resonance imaging. As an alternative, POCUS can facilitate diagnoses at the bedside and ultimately make a significant impact on management decisions.

It is particularly important for clinicians practicing in resource-limited settings to maintain broad differential diagnoses and integrate the POCUS examination with history and physical examination findings and laboratory studies (if available). Point-of-care ultrasound may be the only imaging modality available, and it is therefore crucial to becoming familiar with normal findings, tropical infectious diseases, and some of the rarer pathology seen in these practice settings.

## Case presentation

A 27-year-old female from the Peruvian Highlands presented to the emergency department in Lima with intermittent diffuse abdominal pain and jaundice. There was no reported history of hepatitis or change in stool color. Physical examination revealed scleral icterus and mild tenderness at the right upper quadrant.

**Fig. 1:**
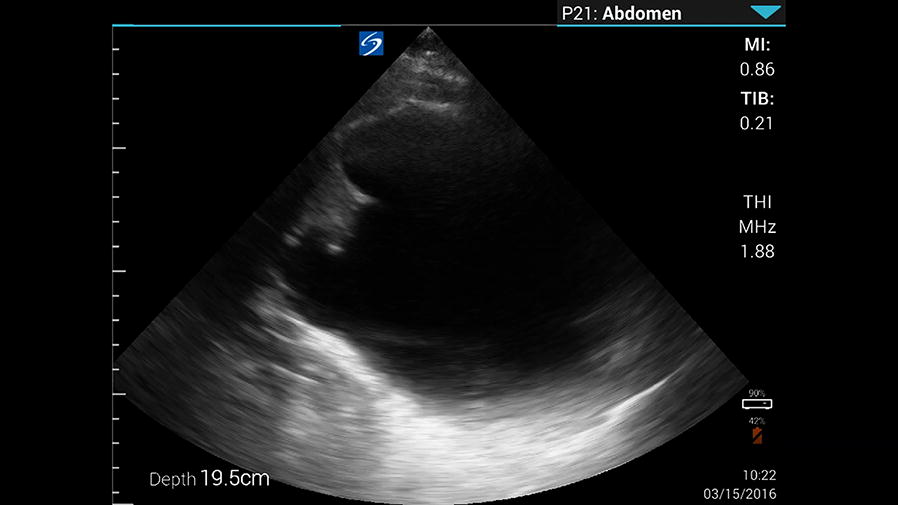
Ultrasound of the patient’s right upper quadrant. There is a large anechoic cystic structure with adjacent smaller cystic structures; these smaller cystic structures are actually the dilated biliary tree. These could easily be confused for daughter cysts that are commonly seen with hydatid cysts of the liver

Given the patient’s region of origin and clinical picture, the initial working diagnosis was a hydatid cyst. A point-of-care ultrasound was performed, which revealed a large multi-locular cyst, with multiple surrounding anechoic structures (Fig. 1, Additional file 1: Video 1). These findings were not typical for hydatid disease, and ultimately the surgical pathology confirmed a choledochal cyst.

## Discussion

**Fig. 2:**
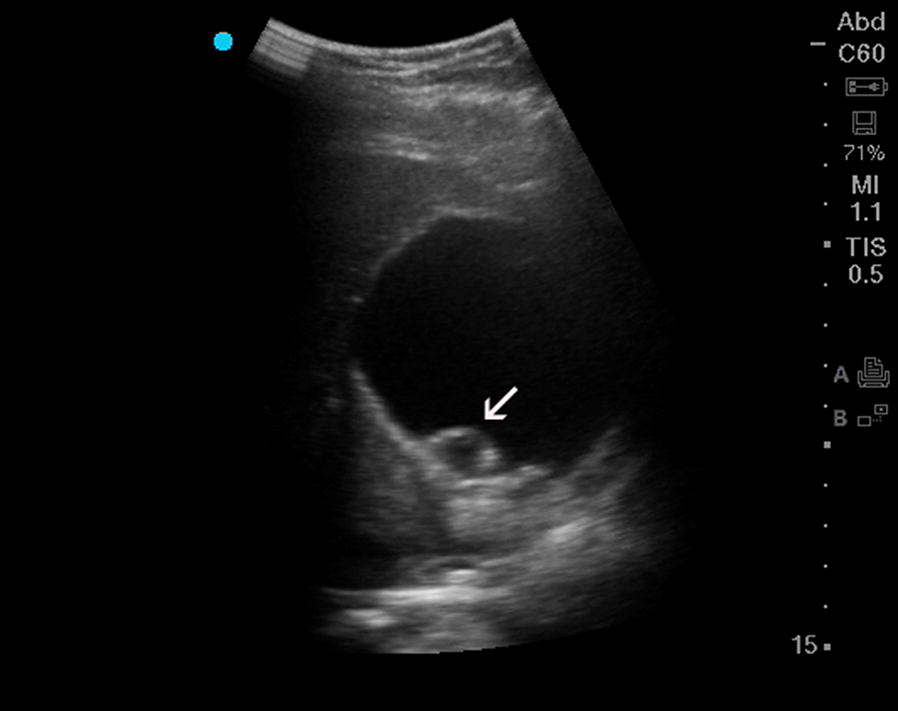
Ultrasound image of an *Echinococcus* cyst. Note the separation of the wall from the cyst (arrow) can be visualized. According to the WHO classification system, this cyst would be considered a type CE2 active cyst [3]

Hydatid cysts (*Echinococcus*) are endemic to the Peruvian Highlands, with a prevalence as high as 9.3% [1]. The hepatic cysts can easily be visualized by ultrasonography (Fig. 2, Additional file 2: Video 2). The first ultrasound classification was described by Gharbi [2], followed by the current World Health Organization (WHO) classification [3].

The patient was taken to surgery for complete excision and was ultimately found to have a choledochal cyst by pathology. The diagnosis of a choledochal cyst is rare, with an incidence of 1/150,000 in Western populations. It is remarkably rare to be diagnosed in adulthood, since approximately 80% of choledochal cysts are diagnosed in infants and children. The most common symptoms at presentation are jaundice and abdominal pain for children and adults, respectively. The most widely accepted treatment is a complete excision, due to the risk of malignant transformation; long-term follow-up and monitoring for malignancy is warranted even after surgical intervention [4, 5].

## Conclusions

This case initially appears as the “classic” finding for *Echinococcus* disease. It is important for the clinician sonographer to appreciate the features consistent with an *Echinococcus* cyst and distinguish it from other pathology. Overall, POCUS can be tremendously useful in the diagnosis and management of patients in the developing world with otherwise limited resources.

## Additional files

**Additional file 1.** Video ultrasound of the patient's right upper quadrant. There is a large anechoic cystic structure with adjacent smaller cystic structures; these smaller cystic structures are actually the dilated biliary tree. These could easily be confused for daughter cysts that are comonly seen with hydatid cysts of the liver.

**Additional file 2.** Video ultrasound of an Echinococcus cyst. Note the separation of the wall from the cyst can be visualized. According to the WHO classification system, the cyst would be considered a type CE2 active cyst [3].

## Abbreviations

POCUS: Point-of-care ultrasound
WHO: World Health Organization
